# Transplantation of fecal microbiota from APP/PS1 mice and Alzheimer’s disease patients enhanced endoplasmic reticulum stress in the cerebral cortex of wild-type mice

**DOI:** 10.3389/fnagi.2022.858130

**Published:** 2022-07-28

**Authors:** Fang Wang, Yongzhe Gu, Chenhaoyi Xu, Kangshuai Du, Chence Zhao, Yanxin Zhao, Xueyuan Liu

**Affiliations:** ^1^School of Clinical Medicine, Weifang Medical University, Weifang, China; ^2^Department of Neurology, Shanghai Tenth People’s Hospital, Tongji University School of Medicine, Shanghai, China; ^3^Shanghai Clinical College, Anhui Medical University, Hefei, China; ^4^School of Nursing, Medical College, Soochow University, Suzhou, China

**Keywords:** FMT, Alzheimer’s disease, ER stress, gut microbiota, TMAO

## Abstract

**Background and purpose:**

The gut-brain axis is bidirectional and the imbalance of the gut microbiota usually coexists with brain diseases, including Alzheimer’s disease (AD). Accumulating evidence indicates that endoplasmic reticulum (ER) stress is a core lesion in AD and persistent ER stress promotes AD pathology and impairs cognition. However, whether the imbalance of the gut microbiota is involved in triggering the ER stress in the brain remains unknown.

**Materials and methods:**

In the present study, fecal microbiota transplantation (FMT) was performed with gut microbiota from AD patients and APP/PS1 mice, respectively, resulting in two mouse models with dysregulated gut microbiota. The ER stress marker protein levels in the cerebral cortex were assessed using western blotting. The composition of the gut microbiota was assessed using 16S rRNA sequencing.

**Results:**

Excessive ER stress was induced in the cerebral cortex of mice after FMT. Elevated ER stress marker proteins (p-perk/perk, p-eIF2α/eIF2α) were observed, which were rescued by 3,3-dimethyl-1-butanol (DMB). Notably, DMB is a compound that significantly attenuates serum trimethylamine-N-oxide (TMAO), a metabolite of the gut microbiota widely reported to affect cognition.

**Conclusion:**

The findings indicate that imbalance of the gut microbiota induces ER stress in the cerebral cortex, which may be mediated by TMAO.

## Introduction

Alzheimer’s disease (AD) is a neurodegenerative disorder that primarily involves the cerebral cortex and hippocampus (Masters et al., 2015). Pathological features of AD include extracellular senile plaques and intra-neuronal neurogenic fiber tangles (NFTs) (Hardy and Higgins, 1992; Niedowicz et al., 2011). Despite many efforts in this field, the pathogenesis of AD remains unclear. In recent years, the role of the gut microbiota in neurodegenerative illnesses has been extensively researched (Bedarf et al., 2017; Wu et al., 2017; Wright et al., 2018). The gut microbiota regulates the function and behavior of the host brain through the brain-gut axis. APP/PS1 mice treated with antibiotics and 3 × Tg AD mice administered probiotics exhibit reduced β-amyloid deposition in the brain (Minter et al., 2016; Bonfili et al., 2017). Furthermore, Aβ pathology can be propagated by transplantation of altered microbiota into sterile APP mice (Harach et al., 2017). Transplantation of gut microbiota from healthy mice attenuates brain pathology as well as memory impairment in ADLP^APT^ mice (Kim et al., 2020). These phenomena involve hypothesized mechanisms mediated primarily by vagal, neuroinflammatory, and gut microbiota metabolites (Ticinesi et al., 2018; Kowalski and Mulak, 2019). Trimethylamine-N-oxide (TMAO), a metabolite of the intestinal microbiota, has recently received increased attention (Brunt et al., 2020; Gatarek and Kaluzna-Czaplinska, 2021). In previous studies, elevated TMAO levels were shown to have a strong clinical correlation with AD and a biomarker for cognitive impairment (Vogt et al., 2018; Wang et al., 2020). Current research indicates that dysbiosis of the gut microbiota affects the onset and development of AD in different ways, rendering the brain-gut axis a promising area for future research.

Increasing evidence indicates that chronic ER stress is highly correlated with cognitive function and sustained stress is considered a pathological driver of AD (Cai et al., 2016). Endoplasmic reticulum (ER) stress markers were generally increased and appeared earlier in brain tissue from AD patients compared with brain tissue from non-demented patients (Hoozemans et al., 2005). ER stress results in the activation of a set of signaling pathways, called the unfolded protein response (UPR), which stimulates specific programs to restore ER function and ensure cell survival (Hetz et al., 2020). However, under prolonged or excessive ER stress, pathological changes in AD are promoted (Oakes and Papa, 2015). The PERK and eIF2α pathways are a branch of the UPR, and their sustained phosphorylation can inhibit synaptic protein synthesis, reduce synaptic plasticity, and exacerbate memory dysfunction (Brewer and Diehl, 2000; Ma et al., 2013; Hetz and Saxena, 2017).

In AD research, a variety of therapeutic approaches to reduce ER stress are under development and mostly include various small molecules that directly inhibit components of the UPR response, with significant efficacy in many preclinical models (Kraskiewicz and FitzGerald, 2012). However, safety aspects are of concern because the UPR has important roles in a variety of cell types and organ physiological states. Therefore, determining what causes ER stress is necessary for regulation at its source. Amyloid-β oligomers can disrupt ER calcium homeostasis, thereby causing ER stress (Gouras et al., 2000). Similarly, tau was shown to increase ubiquitinated protein levels in the brain and trigger activation of the UPR (Abisambra et al., 2013). However, ER stress occurs earlier in AD and may not be caused by amyloid or tau. The brain-gut axis links the brain to the gut microbiota, whether gut microbiota dysregulation causes ER stress in the brain has not been reported, and elucidating the relationship may help identify safe and effective targets for ER stress regulation. Therefore, in the current study, a fecal microbiota transplantation (FMT) approach was used to investigate whether gut microbial dysregulation causes cerebral cortical ER stress and identify the possible mediators involved.

## Materials and methods

### Reagents and antibodies

Ampicillin, metronidazole, neomycin, and vancomycin hydrochloride were purchased from MedChemExpress (Monmouth Junction, NJ, United States). Antibodies against PERK, p-PERK, and p-eIF2α were purchased from Cell Signaling (Danvers, MA, United States), and antibodies against eIF2α and β-tubulin were from Santa Cruz (Dallas, TX, United States). The enhanced chemiluminescence (ECL) kit was purchased from Thermo Fisher Scientific (Waltham, MA, United States). Mouse TMAO Elisa kit was purchased from Kisong Biotechnology (Beijing, China). Hs-CRP Elisa kit was purchased from Elabscience Biotechnology (Wuhan, China), mouse LPS Elisa kit from Cusabio (Wuhan, China), and 3,3-dimethyl-1-butanol (DMB) from Sigma-Aldrich (St. Louis, MO, United States).

### Animals and experimental protocols

Male, 6-week-old C57BL/6J mice were purchased from Shanghai Slac Laboratory Animal Center (Shanghai, China). The mice were given adequate food and water and housed in a laboratory with 24°C temperature, 50–70% humidity, and 12-h light/12-h dark cycles. All animal experiments were performed following the National Institute of Health Guide for the Care and Use of Laboratory Animals and approved by the Animal Ethics and Welfare Committee of Shanghai Tenth People’s Hospital.

Because an antibiotic cocktail was needed to empty the microbes from the mouse gut before FMT, the mice were divided into two groups to evaluate the potential effects of the antibiotic cocktail, the ABX group (*n* = 6) and the control group (*n* = 6). Mice in the ABX group were administered a sterile PBS solution containing an antibiotic cocktail (ampicillin 1 g/L, neomycin 0.5 g/L, vancomycin 0.5 g/L, and metronidazole 1 g/L) by gavage once a day for 3 days in a total volume of 0.2 mL. The control group was not subjected to any intervention. On the 4th day, three mice in each group were sacrificed and the remaining mice were sacrificed on the 17th day after eating a normal diet.

To verify gut microbiota of AD patients and APP mice can cause ER stress in the cerebral cortex of wild-type (WT) mice, all animals were randomly divided into four groups: ABX group, ABX + PBS group, ABX + FMT-APP/PS1 group, and ABX + FMT-AD group. The ABX group was treated with an antibiotic cocktail for 3 days as described in the previous experiment (Bárcena et al., 2019). The ABX + PBS group (*n* = 7) was administered the antibiotic cocktail by gavage for 3 days followed by gavage with PBS containing 20% sterile glycerol three times a week for 2 weeks. The ABX + FMT-APP/PS1 group (*n* = 7) was first administered the antibiotic cocktail for 3 days followed by FMT treatment by gavage for 2 weeks (fecal microbiota of mice in APP/PS1 group). The ABX + FMT-AD group (*n* = 7) was administered the antibiotic cocktail for 3 days followed by FMT treatment (fecal microbiota of AD patients) for 2 weeks.

Furthermore, to verify whether ER stress in the cerebral cortex caused by gut microbial dysbiosis was associated with the gut microbial-associated metabolite TMAO, the mice were divided into six groups: ABX treatment, ABX + DMB treatment, ABX + FMT-APP/PS1, ABX + DMB + FMT-APP/PS1, ABX + FMT-AD, and ABX + DMB + FMT-AD. The duration and dose of the antibiotic cocktail and FMT treatment were the same as described above. DMB treatment was administered to mice from day 4 to day 17 of the experiment with drinking water containing 1% DMB and mice in the other groups were given normal drinking water.

### Fecal microbiota transplantation treatment

Alzheimer’s disease fecal donors were all from the cognitive impairment clinic of Shanghai Tenth People’s Hospital. A total of six AD fecal donors were selected. The mean age of the donors was 72.2 (standard deviation [SD],4.8) years (range 67–80); 50% were women, and 33.3% were APOE ε4 carriers. The mean MMSE score was 18.7 (SD: 1.2). Fresh feces of AD patients and APP/PS1 mice were collected, diluted with sterile PBS solution, vortexed, centrifuged as previously described, and a bacterial suspension was obtained (Sun et al., 2018). Then, the bacterial suspension was mixed with 40% sterile glycerol in an equal volume and stored at −80°C until transplantation (Hamilton et al., 2012). Each recipient mouse was given 200 μL of bacterial suspension Once every 2 days for 2 weeks.

### Elisa

The hs-CRP Elisa kit (Elabscience) was used to measure the serum hs-CRP concentration following the manufacturer’s guidelines, The mouse LPS Elisa kit (Cusabio) and mouse TMAO kit (Kisong) were used to measure LPS and TMAO in serum, respectively, according to the manufacturer’s protocol.

### Western blot

Both cerebral hemispheres were removed from mice after cervical dislocation and stored at −80°C. The frozen cerebral cortex was homogenized in a pre-frozen RIPA buffer. The BCA kit was used to determine the protein concentration of the sample. Total protein (20 μg) was separated using sodium dodecyl sulfate-polyacrylamide gel electrophoresis (SDS-PAGE) and then transferred to polyvinylidene fluoride (PVDF) membranes. Next, the membranes were blocked with 5% BSA solution to inhibit non-specific binding. The membranes were incubated with the following antibodies: rabbit anti-p-PERK (1:1,000), rabbit anti-PERK (1:1,000), rabbit anti-p-eIF2α (1:1,000), mouse anti-eIF2α (1:500), and mouse anti-β-tubulin (1:500). Next, the membranes were incubated for 1 h with the appropriate secondary antibodies (1:2,000). The β-tubulin level was used as the loading control. ImageJ was used to determine the intensity of various molecular bands.

### 16S rRNA sequencing and data analysis in gut microbes

The QIAamp DNA Stool Mini Kit was used to extract microbial genomic DNA from fecal samples following the manufacturer’s instructions. Amplification and sequencing of the 16S rRNA gene were performed as previously described (Huang et al., 2019). The forward primer (338F 5′-ACTCCTACGGGGAGG CAGCA-3′) and the reverse primer (806R 5′-GGACTA CHVGGTWTCTAAT-3′) were used to amplify the V3-V4 region of the 16S rRNA gene (Huang et al., 2019). The sequencing data have been deposited in the Sequence Read Archive (SRA) of the National Center for Biotechnology Information (NCBI) (Bioproject: PRJNA832124) to be released upon publication. QIIME (Quantitative Insights Into Microbial Ecology, v1.8.0)^1^ and the R package 3.5.1^2^ were used for 16S rRNA sequencing analysis. The UniFrac distance metric and primary coordinate analysis were used for diversity analysis to evaluate the structural changes of the microbial communities in the samples (PCoA). LEfSe was used to identify a wide range of taxa. Heat-map was plotted using R package 3.5.1. The relative abundance of gut microbiota was analyzed by one-way ANOVA followed by *post-hoc* comparisons using LSD’s test for multiple groups’ comparisons.

### Statistical analysis

In addition to the detailed description of the 16S rRNA data, one-way ANOVA followed by the least significant difference (LSD) pairwise comparison *post-hoc* test was used for data analysis with GraphPad Prism 9 software. Data were compared between the two groups using a two-tailed Student’s *t*-test. The data are presented as mean ± standard error of the mean (SEM) with a statistical significance of *p* < 0.05.

## Results

### Antibiotic cocktail administration did not affect endoplasmic reticulum stress in the cerebral cortex of wild-type mice

**FIGURE 1:**
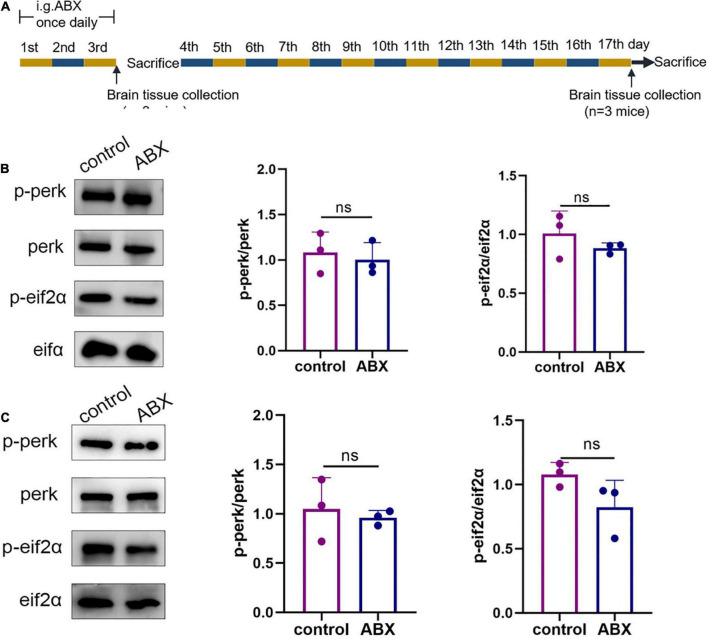
Antibiotic cocktail administration did not affect endoplasmic reticulum (ER) stress in the cerebral cortex of wild-type (WT) mice. **(A)** Experimental design of antibiotic cocktail-treated WT mice (mice were administered an antibiotic cocktail by gavage). **(B)** Representative immunoblots showing p-perk and p-eif2a levels in the cerebral cortex on day 3 of the experiment; *n* = 3 for each group. **(C)** Representative immunoblots on day 17 of the experiment showed p-perk and p-eif2a levels in the cerebral cortex. Data were compared between the two groups using a two-tailed Student’s *t*-test.

Because the microbiota from the mouse gut had to be emptied with the antibiotic cocktail before conducting subsequent experiments, the ABX and control groups were established to examine the possible effects of the antibiotic cocktail on ER stress in the cerebral cortex of WT mice on days 4 and 17 (Figure 1A). Western blotting was used to assess the p-perk/perk and p-eIF2α/eIF2α levels in the cerebral cortex in response to ER stress. On day 4, statistical differences were not observed in the p-perk/perk (ABX group vs. control group, *p* > 0.999) and p-eIF2α/eIF2α (ABX group vs. control group, *p* = 0.7) levels in the cerebral cortex (Figure 1B). Similarly, on day 17, statistical differences were not observed in the p-perk/perk (ABX group vs. control group, *p* = 0.655) and p-eIF2α/eIF2α (ABX group vs. control group, *p* = 0.1) levels in the cerebral cortex (Figure 1C). Therefore, the effect of antibiotic cocktail administration on ER stress in WT mice’s cerebral cortex was excluded.

### Gut microbiome transplantation from Alzheimer’s disease patients and APP/ps1 mice induced endoplasmic reticulum stress in the cerebral cortex of wild-type recipient mice

**FIGURE 2:**
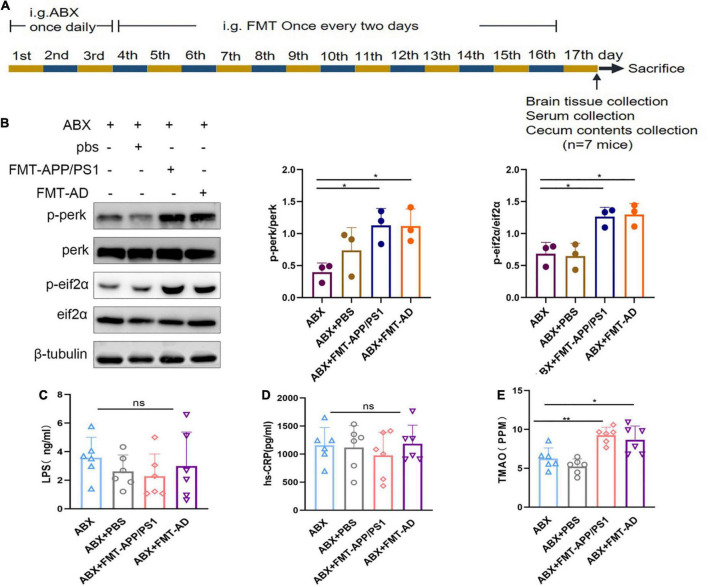
Cortical endoplasmic reticulum (ER) stress marker protein and serum hs-CRP, LPS, and TMAO levels in wild-type (WT) mice after fecal microbiota transplantation (FMT). **(A)** Experimental design of FMT-treated mice. **(B)** Representative immunoblots showing p-perk and p-eif2a levels in the cerebral cortex on day 17 of the experiment. **(C)** Serum hs-CRP levels after FMT. **(D)** Serum LPS levels after FMT. **(E)** Serum TMAO levels after FMT. *P* < 0.05 was set as the threshold for significance by one-way ANOVA followed by *post-hoc* comparisons using LSD’s test for multiple groups’ comparisons, *p < 0.05 and **p < 0.01.

To determine the effect of gut microbiota on ER stress in the cerebral cortex, FMT was performed using fecal bacterial fluid from APP/PS1 mice and AD patients (Figure 2A). After 2 weeks of FMT, the ER stress in the cerebral cortex of mice in the different groups was compared. In the ABX + FMT-APP/PS1 group, p-perk/perk levels were increased (ABX vs. ABX + FMT-APP/PS1, *p* = 0.041) and p-eIF2α/eIF2α levels were increased (ABX vs. ABX + FMT-APP/PS1, *p* = 0.0154). Similarly, in the ABX + FMT-AD group, p-perk/perk levels were increased (ABX vs. ABX + FMT-AD, *p* = 0.045) and p-eIF2α/eIF2α levels were increased (ABX vs. ABX + FMT-AD, *p* = 0.0111). In contrast, p-perk/perk and p-eIF2α/eIF2α levels were not statistically different between the ABX and ABX + PBS groups (Figure 2B). In conclusion, these data indicate that recolonization of the gut microbiota in APP/PS1 mice and AD patients in WT recipient mice can induce ER stress in the cerebral cortex.

### Mice in the ABX + FMT-APP/PS1 and ABX + FMT-Alzheimer’s disease groups did not experience intestinal infection

To exclude FMT failure because mice were in the acute infection phase after FMT which could affect the experimental results, the serum of mice in each group was collected on the last day of the experiment (Figure 2A). Serum hs-CRP and LPS levels were measured using ELISA. The LPS (ABX vs. ABX + PBS vs. ABX + FMT-APP/PS1 vs. ABX + FMT-AD, *p* = 0.595) and hs-CRP (ABX vs. ABX + PBS vs. ABX + FMT-APP/PS1 vs. ABX + FMT-AD, *p* = 0.768) levels were not statistically different between the four groups (Figures 2C,D). Therefore, these results indicate the FMT did not cause acute infection in mice, excluding the possibility that cortical ER stress in mice is due to infection.

### Profiles of intestinal microbiota alterations in the ABX + FMT-APP/PS1 and ABX + FMT-Alzheimer’s disease groups

**FIGURE 3:**
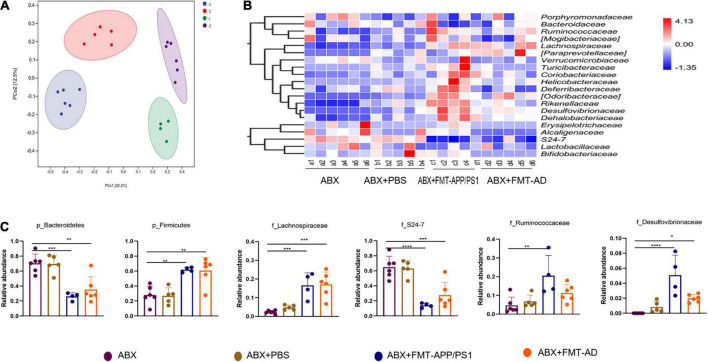
Alterations in the gut microbiota of wild-type (WT) recipient mice after fecal microbiota transplantation (FMT). **(A)** Principal coordinates analysis (PCoA) plots based on unweighted UniFrac distances. **(B)** Alterations in relative abundance (RA) of gut microbiota based on the heatmap. **(C)** RA bar plots of gut microbiota between groups at the phylum and family levels. *P* < 0.05 was set as the threshold for significance by one-way ANOVA followed by *post-hoc* comparisons using LSD’s test for multiple groups’ comparisons, *p < 0.05, **p < 0.01, *** *p* < 0.001, and ****p < 0.0001.

To explore the changes in intestinal microbiota structure in mice after FMT, the intestinal 16s rDNA sequencing results were evaluated and compared with the ABX-treated group, ABX + PBS-treated group, ABX + FMT-APP/PS1 group, and ABX + FMT-AD group. The microbial richness index (Chao1) and diversity index (Shannon) showed no significant difference between the groups (Supplementary Figure 1), indicating the FMT manipulation in this experiment did not affect the richness and diversity of the mouse gut microbiota. However, the UniFrac principal coordinates analysis (PCoA) results showed mice in the ABX + FMT-APP/PS1 and ABX + FMT-AD groups had a significantly different gut microbial composition than mice in the ABX and ABX + PBS groups (Figure 3A).

Therefore, taxonomic changes were compared between taxa, mainly at the level of family or above, as shown in the heatmap (Figure 3B). Specifically, compared with the ABX and ABX + PBS groups, the ABX + FMT-APP/PS1 group showed a significant decrease in the relative abundance (RA) of *Bacteroidetes* (ABX vs. ABX + FMT-APP/PS1, *p* = 0.0004; ABX + FMT-APP/PS1 vs. ABX + PBS, *p* = 0.0007) at the phylum level and *S24-7* (ABX vs. ABX + FMT-APP/PS1, *p* < 0.0001; ABX + FMT-APP/PS1 vs. ABX + PBS, *p* = 0.0002) at the family level, and a significant increase in the RA of *Firmicutes* (ABX vs. ABX + FMT-APP/PS1, *p* = 0.0036; ABX + FMT-APP/PS1 vs. ABX + PBS *p* = 0.0039) at the phylum level and *Lachnospiraceae* (ABX vs. ABX + FMT-APP/PS1, *p* = 0.0018; ABX + FMT-APP/PS1 vs. ABX + PBS, *p* = 0.0006), *Desulfovibrionaceae* (ABX vs. ABX + FMT-APP/PS1, *p* = 0.0009; ABX + FMT-APP/PS1 vs. ABX + PBS, *p* = 0.007), and *Ruminococcaceae* (ABX vs. ABX + FMT-APP/PS1, *p* = 0.0033; ABX + FMT-APP/PS1 vs. ABX + PBS, *p* = 0.0143) at the family level. Furthermore, similar changes were observed in the ABX + FMT-AD group. At the phylum level, the RA of *Bacteroidetes* decreased (ABX vs. ABX + FMT-AD, *p* = 0.0012; ABX + FMT-AD vs. ABX + PBS, *p* = 0.0024) and the RA of *Firmicutes* increased (ABX vs. ABX + FMT-AD, *p* = 0.0018; ABX + FMT-AD vs. ABX + PBS, *p* = 0.0021). At the family level, the RA of *S24-7* decreased (ABX vs. ABX + FMT-AD, *p* = 0.0007; ABX + FMT-AD vs. ABX + PBS, *p* = 0.0018) and the RA of *Lachnospiraceae* (ABX vs. ABX + FMT-AD, *p* = 0.0002; ABX + FMT-AD vs. ABX + PBS, *p* = 0.002), and *Desulfovibrionaceae* (ABX vs. ABX + FMT-AD, *p* = 0.0479; ABX + FMT-AD vs. ABX + PBS, *p* = 0.3996) increased (Figure 3C). Taken together, the above data demonstrate the intestinal microbial characteristics of WT recipient mice after FMT.

### Mice in the ABX + FMT-APP/PS1 and ABX + FMT-Alzheimer’s disease groups showed elevated levels of trimethylamine-N-oxide in serum

Because ER stress occurs in the cerebral cortex after gut microbiota dysbiosis in WT recipient mice, the possible mediators between the gut microbiota and the brain were investigated. Increased production of TMAO (gut microbiota-associated metabolite) has been reported in AD patients and mouse models with gut microbiota dysbiosis. In addition, TMAO can enter the blood and cross the blood-brain barrier. In an *in vitro* TMAO study, TMAO was shown to cause ER stress in cells (Govindarajulu et al., 2020). Therefore, we measured TMAO levels in mouse serum using ELISA. We found that serum TMAO levels were significantly increased in both ABX + FMT-APP/PS1 and ABX + FMT-AD groups of mice compared to the ABX group (ABX vs. ABX + FMT-APP/PS1, *P* = 0.0043; ABX + FMT-AD vs. ABX, *P* = 0.0232) (Figure 2E). These results suggest that TMAO levels in the serum of mice were significantly increased after FMT, and therefore hypothesize that TMAO may mediate the communication between the gut microbiota and the brain.

### Trimethylamine-N-oxide inhibition with 3,3-dimethyl-1-butanol reverses endoplasmic reticulum stress in the cerebral cortex

**FIGURE 4:**
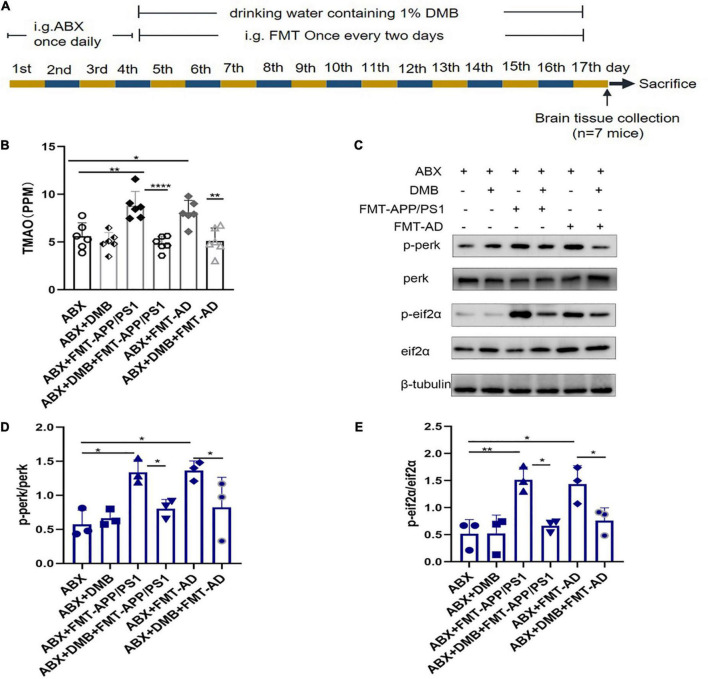
Trimethylamine-N-oxide (TMAO) inhibition with DMB reversed endoplasmic reticulum (ER) stress in the cerebral cortex. **(A)** Experimental design of DMB-treated mice. **(B)** Serum TMAO levels after DMB treatment. **(C–E)**. Representative immunoblots on day 17 of the experiment showed p-perk and p-eif2a levels in the cerebral cortex. *P* < 0.05 was set as the threshold for significance by one-way ANOVA followed by *post-hoc* comparisons using LSD’s test for multiple groups’ comparisons, *p < 0.05, **p < 0.01, and ****p < 0.0001.

To further clarify whether TMAO is a mediator of gut microbiota dysbiosis and ER stress in the cerebral cortex, an additional FMT experiment (Figure 4A) was performed in which the drinking water was supplemented with DMB, which reduces TMAO production by inhibiting microbial TMA lytic enzymes and suppressing plasma TMAO concentrations. The results showed that after DMB supplementation, the TMAO levels in the serum of mice in the ABX + DMB + FMT-APP/PS1 and ABX + DMB + FMT-AD groups were significantly decreased compared to those in the ABX + FMT-APP/PS1 and ABX + FMT-AD groups, respectively (ABX + FMT-APP/PS1 vs. ABX + DMB + FMT-APP/PS1, *P* < 0.0001; ABX + FMT-AD vs. ABX + DMB + FMT-AD, *P* = 0.0029) (Figure 4B). And excessive ER stress occurred in the ABX + FMT-APP/PS1 and ABX + FMT-AD groups, consistent with the previous trend. In contrast, when DMB was administered, ER stress in the cerebral cortex due to gut microbiota dysbiosis was no longer significant in the ABX + DMB + FMT-APP/PS1 group. Similarly, the same effect was observed in the ABX + DMB + FMT-AD group (Figures 4C–E). Thus, gut microbiota dysbiosis-mediated ER stress in the brain may be associated with its effect on increased TMAO production.

## Discussion

The present study findings indicate that FMT of WT recipient mice with fecal microbiota from AD patients and APP/PS1 mice may have increased plasma TMAO levels, which induced excessive ER stress in the cerebral cortex of the recipient mice. The Previous study results showed that FMT in AD model mice using microbiota from the gut of healthy mice improved the cognitive and pathological status of AD mice (Kim et al., 2020). In contrast, FMT of the gut microbiota of patients and model animals in WT recipient mice propagated their pathological features (Harach et al., 2017). Furthermore, transplantation of patient and model animal gut microbiota to WT recipient mice in the present study caused excessive ER stress in the brain cortex of recipient mice by disrupting the structure of their gut microbiota. In addition, the oral TMAO inhibitor DMB had a mitigating effect on ER stress, indicating that TMAO may be a mediator of ER stress in the cerebral cortex caused by dysregulated gut microbiota and TMAO production may be a potential target for AD intervention. The correlation between gut microbiome disorders and AD has been investigated in a variety of clinical and animal studies. Compared with healthy controls, AD patients had reduced gut microbiota diversity, different taxonomic composition, as well as reduced Firmicutes, and increased Bacteroidetes (Vogt et al., 2017). However, in another study, dementia was associated with increased biodiversity, reduced RA of Bacteroidetes, and an increased *Firmicutes*/*Bacteroidetes* ratio (Saji et al., 2019). Notably, in the present study, alterations in the gut microbiota of WT recipient mice caused by transplanting the gut microbiota of AD patients and APP/PS1 mice were similar to those observed in the latter study, with a decrease in RA of *Bacteroidetes* and an increase in RA of *Firmicutes*, in contrast to the observations in the former study, which may be due to a different region and different interventions administered. As mentioned in many studies, excessive ER stress is present in the cerebral cortex of both AD patients and APP/PS1 mice, which can exacerbate amyloid deposition and tau phosphorylation, and in turn exacerbate ER stress (Ma et al., 2013). Notably, in the present study, WT recipient mice showed dysregulated gut microbiota and increased levels of cortical perk and eIF2α phosphorylation, exhibiting excessive ER stress. We hypothesize that dysbiosis of the gut microbiota leads to excessive ER stress in the cerebral cortex.

Furthermore, a 3-day ABX treatment was required before FMT to deplete the gut microbiota, and antibiotic administration significantly alters the gut microbiota composition (Suez et al., 2018) which may persist throughout life. Therefore, we needed to exclude the possible effect of altered gut microbiota caused by ABX treatment on ER stress in the cerebral cortex, thus, mice were sacrificed on days 3 and 14 after ABX treatment to assess ER stress markers (p-perk/perk, p-eIF2α/eIF2α) in the cerebral cortex. The results showed no ER stress in the cerebral cortex of mice sacrificed on days 1 and 14, indicating that antibiotic-induced gut flora depletion did not cause ER stress in the cerebral cortex and the cortical ER stress observed was only associated with recolonization of the gut microbiota in AD patients and APP/PS1 mice.

In the present study, FMT was performed in mice, thus, the recipient mice were at risk of acute infection, and failure of FMT could result in unreliable experimental results. Simultaneously, LPS has been reported to cause ER stress (Sun et al., 2020). Therefore, the serum hs-CRP and LPS levels in the mice were examined after FMT and showed no significant difference compared with the control group, indicating the FMT did not cause acute infection, therefore, the possibility that infection could trigger ER stress in the cerebral cortex was excluded, and LPS was not a mediator of ER stress in the cerebral cortex caused by intestinal microorganisms although LPS is thought to cause ER stress.

The brain-gut axis has recently received increased research attention, in which TMAO (a gut microbiota-associated metabolite) may act as a key signaling mediator (Arrona Cardoza et al., 2021). In a recent clinical study, TMAO was significantly increased in the circulating and cerebrospinal fluid of patients with AD dementia compared with healthy controls (Vogt et al., 2018). In another study, TMAO promoted brain aging and cognitive impairment (Li et al., 2018). Notably, in cardiovascular disease and metabolic disorders, TMAO was shown to induce the ER stress signaling pathway by binding to the ER stress protein PERK (Chen et al., 2019). In human studies, TMAO can be detected in cerebrospinal fluid and elevated in AD (Vogt et al., 2018). Firmicutes and Proteobacteria appear the most active phyla in TMAO production, thus, the imbalance in their relative abundance is often accompanied by an increase in TMAO levels (Romano et al., 2015). In the present study, Firmicutes and Bacteroidetes were significantly increased in the ABX + FMT-APP/PS1 and ABX + FMT-AD groups, thus, TMAO levels may increase and be a key mediator between gut microbes and brain ER stress. Our experimental results did confirm higher TMAO concentrations in the serum of FMT mice, so we designed further experiments when performing FMT by adding the TMAO inhibitor DMB to the drinking water of the mice as previously described (Brunt et al., 2020). ER stress in the cerebral cortex was significantly reduced compared with the control group, indicating that TMAO may play a dominant role in mediating gut microbiota disruption leading to cortical ER stress.

There are some limitations to our study. First, our study used only male mice, so it can only reflect the relationship between gut microbiota dysregulation and ER stress in the male mouse population. Second, the cognitive function of the recipient mice and the pathology of AD in the brain, such as Aβ and p-tau, were not explored further. Therefore, further exploration of these issues is also the next step of our study. Third, extracellular bacterial DNA has been shown to play an important role in protein misfolding (Tetz et al., 2020; Tetz and Tetz, 2021), yet the current study did not analyze whether fecal transplantation increases extracellular bacterial DNA in plasma, so this will be a direction for our future research. Finally, the specific communication pathways between brain and gut microbiota are not fully understood, and how TMAO causes ER stress in the cerebral cortex is unclear, so more in-depth studies are needed to test and verify our findings.

## Conclusion

In summary, the present study results indicate for the first time that host microbiota imbalance may lead to ER stress in the cerebral cortex and ER stress can be rescued by inhibiting TMAO production. In addition, an association was found between gut microbiota and the brain. These results can be used as a basis for reducing ER stress in the brain through modulation of the gut microbiota or its metabolites, contributing to the understanding and treatment of neurological disorders associated with ER dysfunction.

## Data availability statement

The datasets presented in this study can be found in online repositories. The names of the repository/repositories and accession number(s) can be found below: NCBI–PRJNA832124.

## Ethics statement

The studies involving human participants were reviewed and approved by Ethics Committee of Shanghai Tenth People’s Hospital. The patients/participants provided their written informed consent to participate in this study. The animal study was reviewed and approved by Animal Ethics and Welfare Committee of Shanghai Tenth People’s Hospital.

## Author contributions

XL and YZ designed the experiments and revised the manuscript. FW, CX, and YG performed fecal microbial transplantation and drafted the manuscript. FW performed the western blot experiments. KD and CZ performed the analysis of 16srDNA sequencing results. All authors agreed to be accountable for the content of the work.

## Conflict of interest

The authors declare that the research was conducted in the absence of any commercial or financial relationships that could be construed as a potential conflict of interest.

## Publisher’s note

All claims expressed in this article are solely those of the authors and do not necessarily represent those of their affiliated organizations, or those of the publisher, the editors and the reviewers. Any product that may be evaluated in this article, or claim that may be made by its manufacturer, is not guaranteed or endorsed by the publisher.

## Footnotes

1
http://qiime.org/

2
https://www.r-project.org/
