# Supplementary material for: Transplantation of fecal microbiota from APP/PS1 mice and Alzheimer’s disease patients enhanced endoplasmic reticulum stress in the cerebral cortex of wild-type mice
Source: Front Aging Neurosci. 2022 Jul 28;14:858130. doi: 10.3389/fnagi.2022.858130 (PMC9367971; doi:10.3389/fnagi.2022.858130)
Supplement: Supplementary file 1 [file Image_1.pdf]

Supplementary Material

Supplementary Figure 1

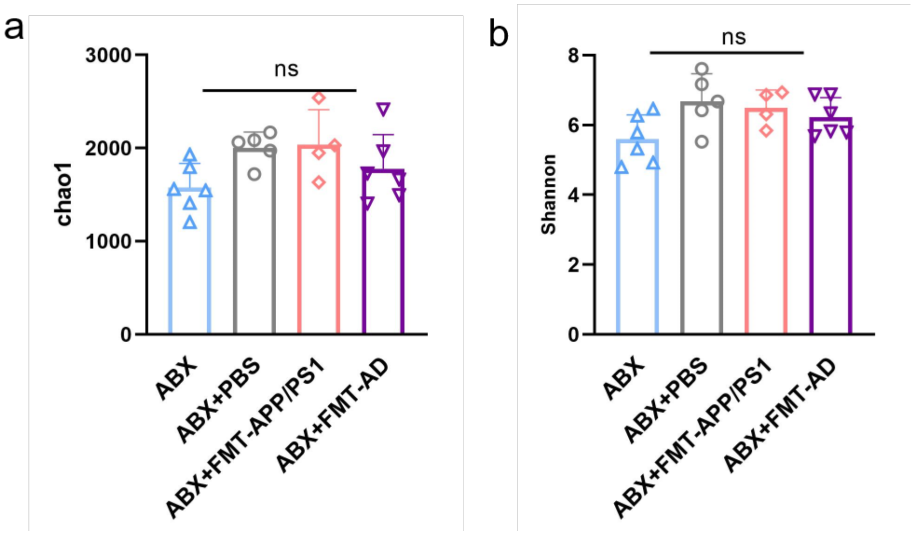

Supplementary Figure 1. The microbial richness index (Chao1) and diversity index (Shannon) of wild-type (WT) recipient mice after fecal microbiota transplantation (FMT). a. Chao1 index in WT mice. b. Shannon index in WT mice.
